# A Comparison of Photodynamic Therapy and Topical Clobetasol in Treatment of Oral Lichen Planus: A Split-Mouth Randomized Controlled Study

**DOI:** 10.3390/jcm14030681

**Published:** 2025-01-21

**Authors:** Jacek Zborowski, Dorota Kida, Bożena Karolewicz, Kamil Jurczyszyn, Tomasz Konopka

**Affiliations:** 1 Department of Periodontology, Wroclaw Medical University, ul. Krakowska 26, 50-425 Wroclaw, Poland; tomasz.konopka@umw.edu.pl; 2 Department of Drug Form Technology, Wroclaw Medical University, Borowska 211 A, 50-556 Wroclaw, Poland; dorota.kida@umw.edu.pl (D.K.); bozena.karolewicz@umw.edu.pl (B.K.); 3 Department of Dental Surgery, Wroclaw Medical University, Krakowska 26, 50-425 Wroclaw, Poland; kamil.jurczyszyn@umw.edu.pl

**Keywords:** oral lichen planus, photodynamic therapy, corticosteroid treatment, new carriers

## Abstract

**Background:** The study aimed to compare the effectiveness of photodynamic therapy (PDT) and topical clobetasol therapy in treating oral lichen planus (OLP). To address the absence of commercially available drug carriers, innovative proprietary solutions were developed. These carriers were designed to enhance the therapies: one for the photosensitizer to reduce its contact time with the mucosa, and another for the steroid to prolong its contact duration. **Methods:** A randomized, single-blind clinical trial lasting three months was conducted on 29 patients with bilateral oral lichen planus using a full contralateral split-mouth design. The authors utilized proprietary carriers containing 5% methylene blue and 0.025%. Lesion size, as well as scores on the Thongprasom, Abisis, and VASs, were assessed during the study. **Results:** Relatively low rates of complete remission of lichen were demonstrated immediately after treatment, 10.3% after PDT and 3.4% after clobetasol, but after 3 months, 79% after PDT, and 62% after CLO. After 3 months of treatment, a reduction of 79.88% for PDT and 56.3% for CLO in the area of the evaluated lesions was achieved. **Conclusions:** PDT emerges as an equally effective method for treating OLP in terms of clinical outcomes, with the added advantage of avoiding many complications associated with conventional therapy.

## 1. Introduction

Oral lichen planus (OLP) is a chronic inflammatory condition primarily affecting the mucous membranes of the mouth. It presents in several distinct clinical forms, each impacting the oral tissue differently. The most commonly recognized types include reticular OLP (the most common type, characterized by white, interlacing lines known as Wickham’s striae), which are erosive [1].

OLP (a severe variant associated with painful ulcers and redness), atrophic OLP (featuring red, atrophic areas), plaque-like OLP (white patches resembling leukoplakia), bullous OLP (less common, presenting with blisters), and papular OLP (often asymptomatic). These variations are crucial for guiding diagnosis and treatment, as more aggressive management and monitoring are required for erosive forms due to the higher risk of malignant transformation [2,3]. Research suggests that the overall risk of malignant transformation ranges from 0.64% to 5.98%, with erosive OLP posing a higher risk (up to 5.98%) compared to non-erosive forms [4].

OLP can manifest both inside and outside the oral cavity, and extraoral involvement may be more prevalent than previously thought. The study showed that around 40% of patients with OLP reported extraoral lesions, with the nails being the most commonly affected site (27.6%), followed by the skin (17.2%), genital mucosa (10.3%), and possible esophageal or pharyngeal involvement (25.3%) [5].

Globally, the prevalence of OLP in the general population is estimated to range from 0.5% to 4%. It is more common in middle-aged adults and occurs more frequently in females than males [6]. Oral lichen planus (OLP) is predominantly a chronic inflammatory condition mediated by T-cells, where immune dysregulation plays a critical role in its pathogenesis. The key immune cells involved include CD8+ cytotoxic T cells, which trigger apoptosis in basal keratinocytes, and CD4+ Th1 and Th17 cells, which release pro-inflammatory cytokines. The immune response in OLP is driven by both antigen-specific and non-specific pathways. Antigen presentation by basal keratinocytes leads to CD8+ T-cell activation, while non-specific mechanisms involve mast cell degranulation and matrix metalloproteinase activation, causing further tissue damage. The STING-TBK1 pathway has also been implicated in OLP, particularly in γδ T cells, which contribute to the production of interferon-gamma (IFN-γ) and interleukin-17 (IL-17), exacerbating inflammation [7]. Additionally, Epstein–Barr virus (EBV) may act as an antigen that triggers immune responses, leading to OLP in genetically predisposed individuals [8]. These immune pathways culminate in chronic inflammation, keratinocyte apoptosis, and mucosal destruction, maintaining the disease’s persistence. Recent research points to the involvement of microorganisms like Helicobacter pylori, Candida albicans, and Mycoplasma salivarium, which may exacerbate immune responses and contribute to disease pathogenesis [9]. Psychological stress, trauma, and systemic diseases, including thyroid disorders and psychiatric conditions, have also been associated with OLP, suggesting a multifactorial etiology [10].

The management of OLP largely centers on reducing inflammation, alleviating symptoms, and lowering the risk of malignancy. Corticosteroids, available in both topical and systemic forms, remain the mainstay of treatment due to their potent anti-inflammatory effects. For mild to moderate cases, topical corticosteroids are preferred, while more severe or treatment-resistant OLP often requires intralesional or systemic corticosteroid therapies [11].

Meta-analyses on the treatment of Oral Lichen Planus (OLP) highlight the efficacy of several therapeutic options, with corticosteroids being the most commonly prescribed treatment. A meta-analysis comparing the efficacy of clobetasol propionate (CLO) with other treatments showed that CLO significantly improved clinical symptoms and reduced lesion size without an increase in adverse events compared to other treatments, making it the most effective and safe first-line treatment for OLP [12].

A systematic review from 2022 analyzing therapies for oral lichen planus (OLP), which included 70 RCT studies with a total of 2612 patients published between 1977 and 2020, confirmed the efficacy of topical corticosteroids as the most effective first-line treatment [13].

Studies indicate that the efficacy of steroids may be limited by the frequency of adverse effects. Approximately 25% of patients report the need to discontinue treatment due to side effects after prolonged use. Moreover, among patients undergoing long-term steroid therapy, there is a tendency for OLP symptoms to recur in 40% of cases within the first two months after discontinuation of treatment. This highlights the need for patient monitoring and consideration of therapy modification if steroid efficacy is insufficient or significant adverse effects occur.

Among corticosteroids, 0.05% clobetasol stands out for its high efficacy in reducing inflammatory and painful symptoms, with studies demonstrating its superiority over other formulations, such as 0.1% triamcinolone and 0.025% fluocinonide. Dexamethasone is primarily used as a rinse solution, making it effective for treating extensive OLP lesions. However, its use requires monitoring due to the risk of fungal superinfections with prolonged applications [13].

Photodynamic therapy (PDT) has become a valuable alternative to corticosteroids in treating Oral Lichen Planus (OLP), particularly in cases where conventional therapies prove ineffective. Research shows that PDT effectively alleviates pain, reduces inflammation, and decreases lesion size in symptomatic OLP. Some studies even suggest that it may surpass topical corticosteroids in efficacy. A systematic review demonstrated notable improvements in both visual analog scale (VAS) scores and lesion size post-PDT, with no adverse effects reported, highlighting its potential as a non-invasive treatment option for OLP [14]. Another study comparing PDT and corticosteroid therapy for erosive OLP found that both treatments significantly reduced pain, although PDT had the advantage of promoting healing without the side effects commonly associated with steroid use [15]. PDT is particularly useful for managing refractory OLP cases, offering a safer, steroid-free option for long-term care in patients unresponsive to other treatments.

However, there is a lack of conclusive evidence supporting the superiority of any one treatment approach, highlighting the need for more research to establish standardized guidelines [16].

Considering the good clinical outcomes and the limitations in the use of corticosteroids, such as allergies or situations where the therapy must be repeated within a short period, PDT (Photodynamic Therapy) emerges as an equivalent therapeutic alternative. A clinical challenge is the method of applying the photosensitizer to the mucous membrane, which affects bioavailability and requires the patient to spend a long time with the agent applied to the membrane. In our study, we proposed a new method of applying the photosensitizer in a mucoadhesive carrier that eliminates these inconveniences.

The study aims to evaluate the effectiveness of traditional clobetasol treatment versus the emerging alternative of photodynamic therapy using methylene blue as the active agent, with both incorporated into porous polymer matrices. These matrices were utilized to modify the contact duration of the active substances with the mucous membrane, shortening it for the photosensitizer and extending it for the steroid. The research focused on comparing reductions in lichenification lesion areas, the progression of the clinical condition, and improvements in patients’ oral health-related quality of life over a three-month follow-up period.

## 2. Materials and Methods

Oral Mucosa and Periodontal Disease Clinic of the Academic Dental Polyclinic of the Medical University of Wrocław. The split-mouth method was chosen to minimize the influence of various confounding factors on the effects of local OLP therapy. Each participant provided informed consent for the study, which was approved by the Bioethics Committee of the Medical University of Wrocław (kb21/2023n) and registered in clinicaltrials.gov with the number NCT06752343.

The sample size was determined using McNemar’s test formula, assuming a type I error rate of 5%, a power of 0.8, a success rate of 0.75 in both groups, and a failure rate of 0.25. A total of 29 patients participated in the study.

Inclusion and exclusion criteria

Patients with bilateral erythematous or erosive OLP lesions larger than 1 cm in diameter, confirmed histopathologically at the Department of Clinical Pathology of the Medical University of Wrocław, were included in the study. The qualification process and assignment of numbers from 1 to 30 were conducted by the same physician.

The main exclusion criterion was dysplasia identified in histopathological examination. Other exclusion criteria included diabetes, liver diseases, smoking (including e-cigarettes with nicotine), pregnancy, or objective determination of a lichen planus-like nature of the lesions.

Carriers with clobetasol and methylene blue.

The method of preparation and the composition of carriers for the active substances used in the 2024 study were identical to those in the authors’ initial pilot study from 2022 [17]. The matrices were obtained by lyophilizing foam formed from a dispersion of pullulan, sodium alginate, and methylcellulose. The active substances, clobetasoli (PolAura) or methylene blue (abcr, Karlsruhe, Germany), were added to the homogeneous mixtures (Table 1).

**Figure 1:**
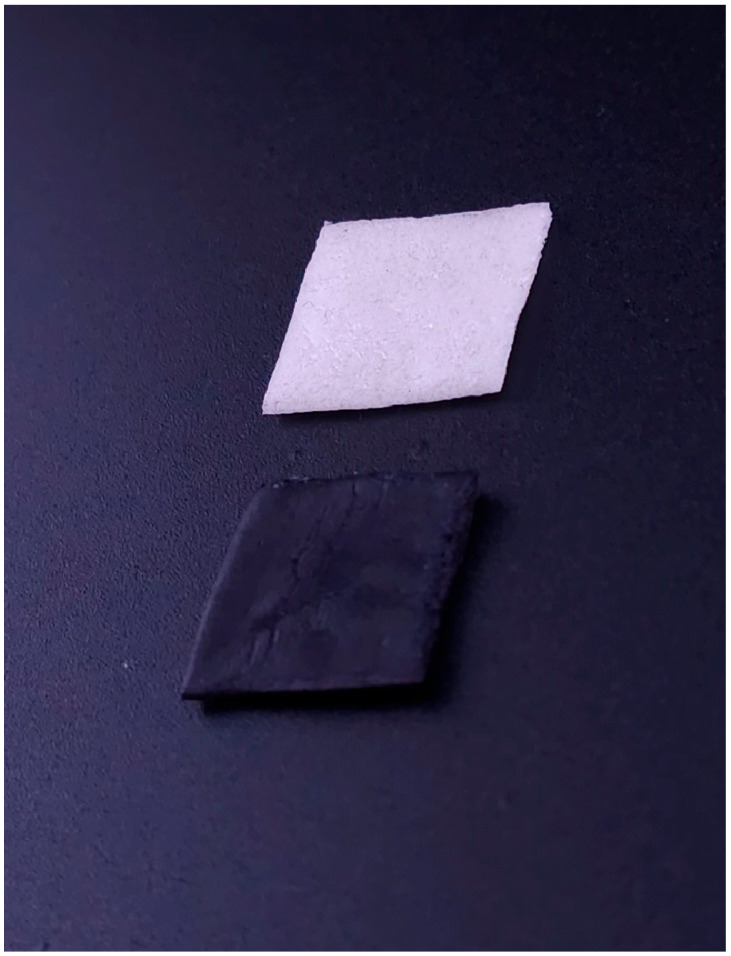
Carrier with methylene blue (**lower**) and clobetasol (**upper**) before application.

The developed carriers containing clobetasol and methylene blue (Figure 1) demonstrated high mechanical strength, smear resistance, and effective release of active substances during in vitro tests. The carrier was precisely fitted to the size of the treated lesion on the mucosa, allowing accurate determination of the active substance amount in mg/cm^2^ in the form of the dry carrier [17].

Study protocol

The study protocol was identical to the authors’ previous study [17].

**Figure 2:**
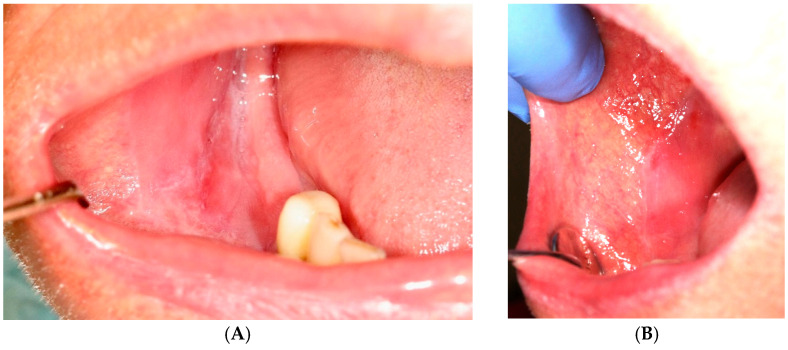
Oral lichen planus before (**A**) and after (**B**) photodynamic therapy.

**Figure 3:**
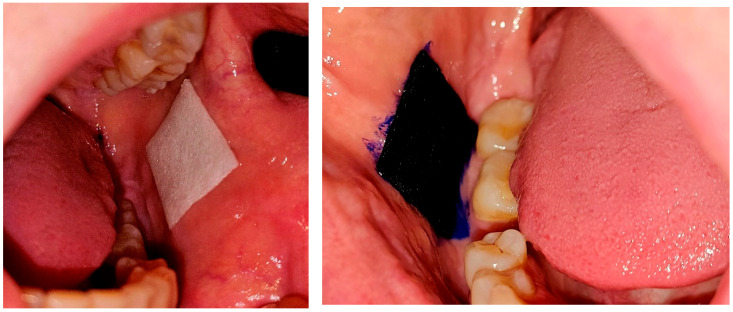
Carrier with methylene blue (**left**) and clobetasol (**right**) cut to the lesion size and applied on the mucosa.

PDT (photodynamic therapy) was performed four times on days 1, 3, 6, and 9 (Figure 2). Following a 10 min application of a carrier with 5% methylene blue, activation was carried out using a diode laser with a wavelength of 650 nm. (Figure 3) The procedure was performed by the same clinician using an energy density protocol of 120 J/cm^2^ and a power of 1034 mW/cm^2^ for 227 s.

**Figure 4:**
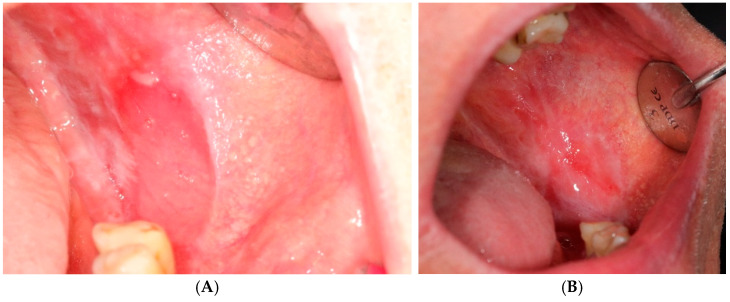
Oral lichen planus before (**A**) and after (**B**) clobetasol therapy.

Steroid therapy was conducted over nine days, with the application of a carrier fitted to the required size on the other OLP lesion (Figure 4). The procedure was performed by the clinician after each PDT session, while on days 2, 4, 5, 7, and 8, patients applied the carrier themselves (Figure 3). Randomization involved assigning the therapy according to the order of enrollment: patients with even numbers received PDT on the left side and steroids on the right side, while odd-numbered patients received the reverse.

**Figure 5:**
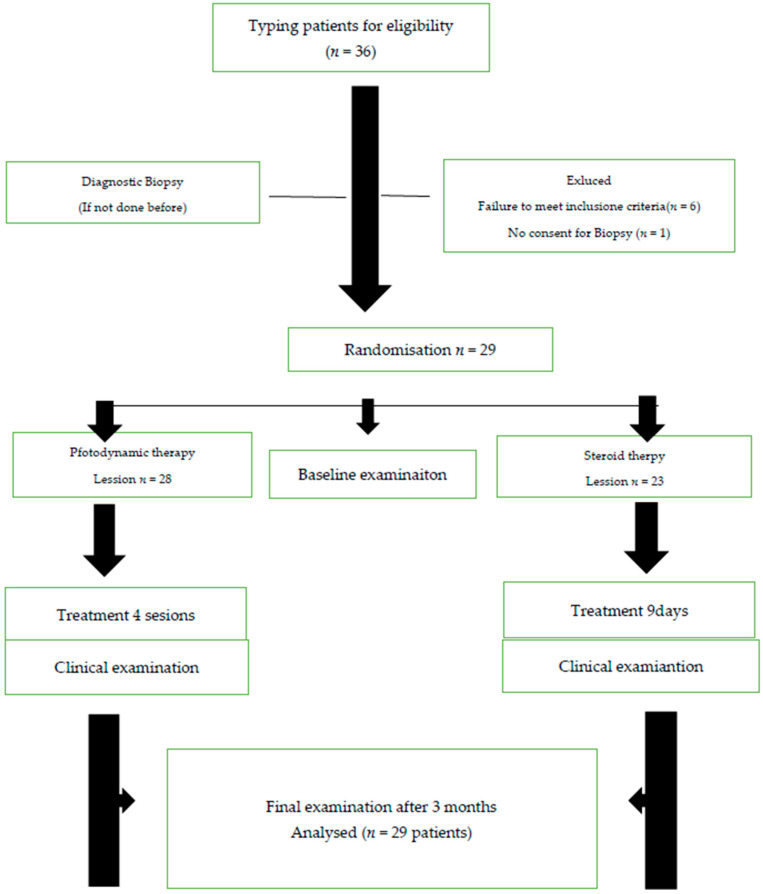
Flow diagram.

Clinical evaluation of OLP (measuring the lesion area in mm) was performed using a PCP UNC15 periodontal probe (Hu-Friedy, Chicago, IL, USA) on the day of study initiation, at the end of the active phase, and after 90 days. Patients also reported pain levels using a visual analog scale (VAS) from 0 to 10, while oral health-related quality of life (OHRQoL) was assessed using the OHIP questionnaire. Additionally, the Autoimmune Bullous Skin Intensity Score (ABSIS) [18] was evaluated at the beginning and end of the study according to the protocol outlined above (Figure 5).

The progress of OLP therapy was monitored using the Thongprasom scale [19], while treatment efficacy was assessed according to the protocol by Carrozzo and Gandolfo [20].

For improvement on the Thongprasom scale (from 4 to 0, with no improvement or worsening observed in only 3 patients), the most statistically significant multiple regression model indicated a significant effect of the number of teeth and initial VAS value, with no significant effect of treatment type. The multiple regression equation was:

Change in Thonga’s scale = 3.53 − 0.05 number of teeth − 0.09 initial VAS ± 0.88

## 3. Result

Table 2 summarizes the demographic data of 29 patients and the clinical parameters evaluated for the entire group, as well as those treated with both protocols for red lesions on the oral mucosa. The majority of the study group consisted of women, with a mean age of 65 years. The OLP lesions observed in the participants were more advanced forms, confirmed by histopathological examinations, and typically had a history of several years.

The primary location of the lesions was the buccal mucosa, presenting as red or erosive lesions in 23 patients. Only one patient had a plaque-like form located on the tongue, while four had desquamative lesions located on the gingiva. Lesions treated with photodynamic therapy were statistically larger. The assessment of pain intensity, measured using the VAS, revealed significant heterogeneity.

In nine patients, lichen lesions outside the oral cavity were identified, but they did not require treatment. This includes the relationship between the oral health status of individuals treated for lichen planus and their quality of life. Pain, particularly during eating, has the greatest impact on the quality of life of patients with oral lichen planus (OLP), as assessed by OHRQoL (Oral Health-Related Quality of Life). This is reflected in the average VAS scores exceeding 5.5 on a 10-point scale, as well as OHRQoL parameters in the domains of physical pain (4.07 ± 1.7) and psychological discomfort (3.07 ± 1.9).

No significant differences were observed in the analyzed clinical parameters of lesions qualified for treatment on both sides of the oral cavity, except for the larger area of lesions treated with PDT (photodynamic therapy). Most patients exhibited significant (+++) or moderate (++) inflammation intensity as determined by histopathological examination.

Table 3 presents the effectiveness parameters of both treatment methods for OLP (oral lichen planus) based on the Carrozzo and Gandolfo scale. The vast majority of patients showed partial improvements immediately after therapy; however, during the 3-month follow-up period, this parameter significantly improved. Complete remission of the disease occurred as follows: immediately after treatment, 10.3% for PDT and only 3.4% in the clobetasol (CLO) group; after three months, 79% for PDT and 62% for CLO. Notably, there were significantly more cases of full OLP remission, according to the Carrozzo and Gandolfo scale, observed following both therapeutic approaches. These results provided stronger statistical evidence, allowing for the rejection of the hypothesis of no clinical improvement three months after either treatment method, with an advantage favoring the PDT method. However, one patient in the PDT group did not respond to treatment, whereas no such cases were observed in the Steroid group.

Table 4 presents the changes in lesion area and the Thongprasom scale scores for OLP lesions treated with both methods. Initially, a larger lesion area was observed in the PDT-treated group. The presence of red OLP lesions in the oral cavity had the greatest impact on OHRQoL (oral health-related quality of life), primarily due to pain and psychological discomfort.

Assessment of the lesion area on the mucosa

Compared to baseline results, a significant reduction in lesion area was noted following both PDT and topical steroids during all subsequent observations. After 3 months of treatment, the lesion area decreased by 79.88% for PDT (Figure 2) and 56.3% for CLO (Figure 4). Clinical improvement, assessed using the Thongprasom scale, showed a transition of red, erosive lesions to white forms. Although erythema was frequently observed immediately after treatment, during the 12-week follow-up, this fraction significantly decreased, with more pronounced effects in remission achieved through photodynamic therapy. Almost all parameters demonstrated statistical significance when compared across the three evaluations.

Assessment of Quality of Life

The applied oral cavity therapy did not affect the presence of skin lesions when co-occurrence with mucosal changes was observed (Table 2). After the 3-month therapy, a significant reduction in the size of OLP lesions was noted, as indicated by the second domain of the ABSIS scale. There was a substantial reduction in pain, as assessed by patients using the VAS, ABSIS3, and the physical pain domain of OHIP-14, both immediately after treatment and three months later. The therapy significantly impacted most evaluated parameters, including quality of life measured using OHIP-14, as well as other domains of the OHQRoL questionnaire, such as psychological discomfort, physical, and psychological limitations (Table 5).

Validation and Analysis of Statistical Models for OLP Outcomes

The model validation data are as follows: F = 4.09, *p* = 0.028, R = 0.49, and R^2^ = 0.24, partial regression coefficients for number of teeth −0.35 (*p* = 0.049), and initial VAS −0.34 (*p* = 0.048), no multicollinearity between independent variables (tolerance 0.99, R^2^ = 0.005), homoscedasticity based on residuals versus predicted values (uniform scatter plot), residual autocorrelation (Durbin-Watson test d = 1.65, R = 0.15), normal distribution of residuals based on residuals versus normal value scatter plot, and mean Cook’s distance of 0.04.

For OLP remission according to the modified Carrozzo and Gandolfo scale in the 3-month post-treatment observation (dichotomous variable: remission in 41 cases, no remission or worsening in 10 cases), the logistic regression model revealed a significant impact of initial lesion area (smaller areas favored remission) and location (lesions on the buccal mucosa—code 3—were more likely to remit compared to lesions on the tongue—code 2—or gingiva—code 1). The model also required the inclusion of the initial lesion description in the Thongprasom scale. The logistic regression equation was:

Logit P = −4.615 − 0.006 initial lesion area + 0.91 lesion location codes

The model validation data are as follows: Chi^2^ = 8.81, *p* = 0.031; OR for lesion area 0.994 (0.989–0.999) and location codes 2.49 (1.0–6.2). The significance of regression coefficients are as follows: lesion area *p* = 0.03, lesion location codes *p* = 0.049, normal distribution of residuals based on residuals versus expected normal value scatter plot, and mean residual value of 0.07.

No significant correlation was found between any of the analyzed baseline clinical variables and OLP improvement on the Thongprasom scale in the final observation.

## 4. Discussion

Steroid therapy plays a key role in the treatment of oral lichen planus (OLP) and is considered the gold standard in therapeutic management. Corticosteroids, such as clobetasol and triamcinolone, are widely used due to their potent anti-inflammatory effects, which alleviate symptoms such as pain and ulceration. In topical therapy, corticosteroids effectively reduce inflammation, improve patients’ quality of life, and minimize disease progression [1,2,21,22].

However, their use is associated with significant risks of side effects. The most common adverse effects include thinning of the mucous membrane, which increases susceptibility to injuries, as well as secondary infections such as oral candidiasis. Patients may also experience burning, dryness, or hypersensitivity at the application site, and there are limitations on the rapid repetition of therapy [23,24,25].

For this reason, alternative treatments for oral lichen planus are being explored. One such option is photodynamic therapy (PDT), which has demonstrated comparable clinical efficacy to corticosteroids as a first-line treatment. Clinical studies have shown that PDT leads to significant reductions in pain and inflammation in 75% of patients within three weeks of therapy, which is comparable to the effectiveness of clobetasol [26].

Meta-analyses have demonstrated that PDT reduces lesion size by 1.53 cm^2^ (95% confidence interval: 0.71–2.35), with a partial response (PR) rate of 0.77 (95% CI: 0.65–0.85). Scores on the Thongprasom scale decreased by 1.33 (95% CI: 0.56–2.10), while pain scores on the VAS were reduced by 3.82 points (95% CI: 2.80–4.85). During the procedures, patients reported only mild side effects, such as a burning sensation, which resolved immediately after PDT sessions [27]. Importantly, patients undergoing PDT experienced a lower risk of relapse compared to those treated with steroids, where OLP symptoms recurred in approximately 40% of patients within two months after discontinuing treatment. PDT also reduces the risk of adverse effects associated with prolonged corticosteroid use, such as mucosal atrophy and fungal infections, making it particularly advantageous for patients who cannot tolerate long-term steroid therapy [28].

Moreover, although the cost of PDT is higher than standard steroid treatment, single-session therapies may offer a better cost–benefit ratio for the chronic nature of OLP, reducing the need for repeated visits and continuous medication use. These findings suggest that PDT may serve as an effective and safe alternative for patients with OLP, especially in cases resistant to traditional steroid therapy or when long-term corticosteroid use is contraindicated [29].

PDT also has its challenges. Regardless of the photosensitizer used, the method of application and the required contact time between the photosensitizer and the mucosal lesion present significant obstacles. These factors affect the bioavailability of the agent and the absorption process in the oral mucosa, influenced by the oral cavity environment, which directly impacts the effectiveness of the treatment. Therefore, in our study, a novel approach was proposed in the form of a mucoadhesive carrier incorporating the active agent. Similar conclusions regarding the need for a new carrier were drawn by the authors of a multicenter study published in 2022. In this study, 122 patients were treated using a mucoadhesive carrier with clobetasol designed specifically for therapy, named Rivelin^®^-CLO (20 μg) [30].

The use of custom polymer matrices enabled the conduction of randomized controlled trials (RCTs) using the “split-mouth” design. This design minimizes the influence of confounding etiological factors. Additionally, the “split-mouth” model requires fewer participants than a parallel randomization scheme but poses greater challenges in recruiting patients, as OLP lesions must be bilateral and of comparable extent. Patients were enrolled in the study solely based on histopathological diagnosis and the presence of the red clinical variant of OLP.

In a previous study, the authors used the same carrier, but with triamcinolone as the steroid. The activation of the photosensitizer was carried out using a custom-designed laser device with a wavelength of 630 nm. The study achieved partial response (PR) rates of 33.3% with PDT and 22.2% with triamcinolone (TA) immediately after treatment, and after 3 months, 54.2% with PDT and 62.9% with TA [17]. The choice of photosensitizer for therapy was guided by the comfort of both the patient and the practitioner, due to its short application time of 10 min (significantly shorter than 5-ALA), which makes this therapy feasible in any outpatient setting.

A 2020 meta-analysis assessed the outcomes of oral lichen planus (OLP) treatment using various photosensitizers (PS), such as aminolevulinic acid (5-ALA), methylene blue (MB), and chlorin e6. A 5% concentration of 5-ALA, kept in contact with the lesion for 30 to 120 min, demonstrated high efficacy, achieving a partial response (PR) in 86% of cases. In contrast, methylene blue showed lower efficacy, which may have been due to its shorter application time (typically 5 min as a rinse) [31].

In this study, clobetasol (CLO) at a 0.25% concentration was selected as one of the most effective and frequently used steroids in the topical treatment of OLP and was compared with PDT using 5% methylene blue delivered in a mucoadhesive form.

The efficacy of this steroid was evaluated, among others, by Sivaraman in 2016 in a randomized, triple-blind study. He compared clobetasol 0.05% with triamcinolone 0.1% and tacrolimus 0.03%, demonstrating that clobetasol had the highest efficacy, while tacrolimus showed the lowest, with three times less complete lesion resolution compared to clobetasol [32].

In a 2022 study encompassing 35 RCTs and 1474 patients treated with steroids and calcineurin inhibitors, Lodi recognized steroids as first-line drugs. However, he highlighted the significant heterogeneity of the results obtained and the lack of clear conclusions regarding both the efficacy of the therapy and its side effects [13].

The literature reports various concentrations of clobetasol used in treatment. This issue was addressed in studies by Carbone (2009) and Campisi (2004), which compared the efficacy of different formulations and concentrations of clobetasol ointments used in OLP treatment. Carbone compared 0.025% and 0.05% concentrations, finding no significant difference in outcomes (RR 1.14, 95% CI 0.56 to 2.35). Similarly, Campisi examined the effects of 0.025% clobetasol microspheres compared with the standard formulation of the same concentration, also noting no significant differences between patient groups [33,34,35,36].

The carriers used in our study were additionally laminated on the oral cavity side to ensure that the therapeutic agent’s activity was directed only toward the mucosa. In the case of methylene blue, this lamination also minimized “staining” of the oral cavity. Our results demonstrate that PDT is a very good alternative to classical steroid therapy in terms of lesion regression, as evaluated by the Thongprasom (TH) scale [37], achieving a score of 1.30, comparable to corticosteroids. Yiemstan, based on a study involving 69 patients, concluded that a change in clinical category from 2 to 3 on the TH scale, indicating an increase in atrophic area, worsened quality of life [38]. Our clinical observations align with results obtained by other authors. Levee, in his study evaluating the photosensitizer toluidine blue, achieved a reduction in the TH parameter by −1.87 ± 1.457 [39].

In our study, the complete response (CR) rate at 3 months was 79% for PDT and 62% for CLO. After 3 months of therapy, the lesion area decreased by 79.88% for PDT and 56.3% for CLO. The observations made by the authors align with many studies and are often even better. In Mostafa’s study, 37% of lesions treated with steroids and 5% of lesions treated with PDT did not respond to therapy at all. The results of this study also indicate greater clinical effectiveness of OLP treatment with PDT compared to topical steroid therapy, primarily due to its superior efficacy in relieving pain and reducing lesions [40].

Sulewska and colleagues [41] demonstrated that weekly PDT sessions over 10 weeks resulted in an 8% reduction in lesion size immediately after the procedure and a 67% reduction after 12 months of observation. This study utilized a non-standard LED lamp with an intensity of 150 J/cm^2^ and 5% 5-aminolevulinic acid as the photosensitizer.

In the authors’ study, mucocutaneous lichen planus was diagnosed in 30% of patients. The mean ABSIS1 index for skin lesions was low and did not show significant changes following topical OLP treatment. Bilateral topical treatment in patients resulted in a significant reduction in pain intensity, particularly immediately after therapy. Pain was assessed using the visual analog scale (VAS), decreasing from 5.72 ± 2.3 at the start of the study to 2.62 ± 1.7 after therapy, indicating a 3.1-point reduction with statistical significance (*p* < 0.000). This is also supported by the results of the discomfort index during food consumption (ABSIS3), which showed a 3.0-point reduction (*p* < 0.000), as well as the physical pain domain of the OHIP-14 questionnaire, which demonstrated a 1.66-point difference.

The assessment of (OHRQoL) is an extremely important aspect of research, as it negatively affects daily functioning, including social relationships, and results in physical and psychological consequences [42,43].

Studies by Alves have shown that patients with OLP are more likely to experience anxiety, depression, and other negative consequences of the disease that impact their lives. The researchers suggest that a better understanding of how symptomatic OLP affects psychological well-being could enable clinicians to implement more personalized and effective treatments, combining mucosal therapy with psychological care [44].

The impact of OLP is more negative in the red forms, particularly those involving epithelial disruption. In our study, the total reduction in OHIP-14 scores after completed therapy was 5.51 points (*p* < 0.000), with the most significant improvements observed in the domains of physical pain, psychological discomfort, and psychological limitations.

In Saberi’s study, quality of life was assessed using the Chronic Oral Mucosal Disease Questionnaire (COMDQ), and pain was measured using the VAS. The study included 60 patients with the erosive-ulcerative form of OLP. A significant correlation was found between oral pain and the total COMDQ score, as well as its physical, social, and emotional domains, in patients with erosive/ulcerative OLP [43].

Improvement in OHRQoLwas significantly associated with reductions in pain and clinical symptom severity. These findings are corroborated by Ketonga’s study, which evaluated 72 patients undergoing topical steroid therapy [45].

On the other hand, Parlatescu’s 2020 study of 160 patients (80 in the study group and 80 in the control group), demonstrated that the most common keratotic OLP (48.75% of participants)—did not significantly impact quality of life. However, a negative social impact was observed in the psychological discomfort domain compared to the control group [46].

Patients tolerated the therapy well, reporting only minor side effects commonly mentioned in the literature, such as mild irritation or burning immediately after photodynamic therapy (PDT), clinically manifested as localized redness [41,47]. Other reports, such as Lavaee’s study, noted no adverse effects at all, even minor ones, during therapy [39]. Conversely, Sulewska reported pain during PDT in participants with depression or recurrent OLP [41].

## 5. Conclusions

Our study does, of course, have its limitations—the main ones being the relatively small study group and the simple method used to measure lesion size. Such an assessment is possible under non-visible light, which may affect the readings. Additionally, the choice of the split-mouth method, while minimizing confounding factors, does not eliminate them entirely.

Considering the cited studies, including meta-analyses and reviews, PDT emerges as an equally effective method for treating OLP in terms of clinical outcomes, with the added advantage of avoiding many complications associated with conventional therapy. The results of the authors’ own studies indicate a very high efficacy of PDT in the topical treatment of oral lichen planus.

A critical requirement for PDT is thorough diagnosis and histopathological evaluation before initiating therapy. The authors advocate for the use of mucoadhesive dressings for administering photosensitizers and lasers as light sources. However, longer clinical follow-ups are necessary to conclusively determine the value of PDT in OLP treatment.

The authors suggest that PDT should be considered equivalent to steroid therapy or recognized as an effective alternative in OLP treatment guidelines.

## Figures and Tables

**Table 1:** Composition of matrices with clobetasol (CLO) and methylene blue (MB).

Formulation Code	Composition [% in Dry Mass]				
	Pullulan	Sodium Alginate	Glycerol	Methylcellulose	Active Substance
CLO	36.6	9.7	48.6	4.85	0.25
MB	27.0	7.3	38.9	4.3	22.5

**Table 2:** The baseline demographic and clinical data of enrolled patients and sites with OLP.

Variables	Patients (29)	Treatment Lesions	
		PDT (28)	Steroid (23)
Age: interval, mean (SD)	37–87, 64.5 (10.6)	64.3 (10.1)	64.7 (11.6)
Gender: F/M (n)	24/5	24/4	20/3
Intensity of inflammation in histopathological examination (n)	+ 2 ++ 13 +++ 14		
OLP duration in years: interval, mean (SD)	2–15, 5.5 (3.5)	5.5 (3.4)	5.6 (3.7)
Number of tooth: median	0–28, 21	21	21
ABSIS1: No > 0, mean (SD)	9, 0–81, 6.6 (16.8)		
ABSIS2: interval, median	2–9, 4		
ABSIS3: No > 0, mean (SD)	25, 0–16, 6.1 (5.4)		
VAS: interval, mean (SD)	1–10, 5.72 (2.3)		
OHIP-14: interval, mean (SD)	4–26, 14.1 (6.8)		
Location of treated OLP lesion		Buccal mucosa: 23 Gingiva: 4 Tongue: 1	14 8 1
Lesion size in cm^2^: mean (SD)		24.9 (24.7)	20.0 (13.5)
Thongprasom score: interval, median, score (n)		3–5, 3 Score 3–21 Score 4–3 Score 5–4	3–5, 3 Score 3–19 Score 4–2 Score 5–2

**Table 3:** Evaluation of treatment results according to modified Carrozzo and Gandolfo scores; CR—complete response and PR—partial response.

Type of Treatment	Immediately After Treatment			3 Months After Treatment		
	CR	PR	No Response	CR	PR	No Response
PDT	3	21 ^A^	4	23 ^C^	4	1
ST	1	20 ^B^	2	18 ^D^	5	0

A—*p* < 0.0001; B—*p* < 0.0001; C—*p* < 0.0001; D—*p* = 0.0001.

**Table 4:** Changes in mean lesion size and Thongprasom score for OLP lesions in both treatment methods.

Lesions Clinical Scores	Treatment Method	Baseline	Immediately After the End of Treatment	After 3 Months Follow-Up	*p* Values
Lesion size in cm^2^	PDT	24.94 ± 24.7	10.09 ± 11.1	5.02 ± 6.0	1 vs. 3 *p* < 0.000
	ST	20.02 ± 13.5	9.84 ± 10.2	8.75 ± 18.6	1 vs. 3 *p* < 0.000
Thongprasom score (means and medians)	PDT	3.39 ± 0.7, 3	2.18 ± 0.9, 2	1.29 ± 0.8, 1	1 vs. 3 *p* < 0.000 2
	ST	3.26 ± 0.6, 3	2.04 ± 0.8, 2	1.3 ± 0.6, 1	1 vs. 3 *p* < 0.000 2

**Table 5:** Changes in clinical variables and oral health related quality of life for OLP patients due to the treatment taken.

Variables in Patients	Baseline	Immediately After the End of Treatment	After 3 Months Follow-Up	*p* Values
ABSIS1 (No > 0, means)	9, 6.59 ± 16.8	ND	9, 5.66 ± 16.2	1 vs. 3 *p* = 0.18
ABSIS2 (median)	4	2	2	1 vs. 3 *p* < 0.000 2
ABSIS 3 (means)	6.1 ± 5.4	ND	3.1 ± 3.6	1 vs. 3 *p* < 0.000
VAS (means)	5.72 ± 2.3	3.38 ± 1.6	2.62 ± 1.7	1 vs. 3 *p* < 0.000 2
Overall OHIP-14	14.1 ± 6.8	ND	8.59 ± 4.9	1 vs. 3 *p* < 0.000
Functional limitations	1.86 ± 1.9	ND	0.86 ± 1.0	1 vs. 3 *p* = 0.003
Physical pain	4.07 ± 1.7	ND	2.41 ± 1.5	1 vs. 3 *p* < 0.000
Psychological discomfort	3.07 ± 1.9	ND	2.0 ± 1.4	1 vs. 3 *p* = 0.006
Physical disability	2.17 ± 1.7	ND	1.41 ± 1.6	1 vs. 3 *p* = 0.03
Psychological disability	1.72 ± 1.4	ND	0.87 ± 1.3	1 vs. 3 *p* = 0.01
Social disability	0.72 ± 1.2	ND	0,72 ± 1.4	1 vs. 3 *p* = 0.9
Handicap	0.41 ± 0.9	ND	0.31 ± 0.8	1 vs. 3 *p* = 0.67

## Data Availability

The raw data supporting the conclusions of this article will be made available by the authors on request.
